# The main predictors of well-being and productivity from a gender perspective

**DOI:** 10.3389/fpsyg.2024.1478826

**Published:** 2024-11-07

**Authors:** Kevin Martínez-Martínez, Susana Llorens, Valeria Cruz-Ortiz, Juanjo Reyes-Luján, Marisa Salanova

**Affiliations:** WANT Research Team, Department of Social Psychology, Universitat Jaume I, Castellón, Spain

**Keywords:** gender, resources, demands, practices, well-being, performance

## Abstract

**Introduction:**

Gender difference management is one of the most challenging dimensions organizations must cope with to adapt to VUCA (Volatile, Uncertain, Complex and Ambiguous) environments. The aim of this study is to identify the main drivers (i.e., most influential job resources, job demands, and organizational practices) of healthy employees and organizational outcomes assessing the differences between men and women, based on the HERO (HEalthy and Resilient Organization) Model.

**Methods:**

Data were collected through the HERO-CHECK Questionnaire: job demands (e.g., quantitative overload), job (e.g., team coordination) and personal resources (e.g., emotional competence), healthy organizational practices (e.g., work-family balance practices), healthy employees (e.g., work engagement) and healthy organizational outcomes (e.g., in-role performance). The sample consisted of 2,128 professionals (70% female) from 8 organizations. Gender-based multigroup SEM was performed using R 4.1.2.

**Results:**

Results of the multigroup SEM analysis show a good fit of the HERO model and support the existence of configural invariance among gender groups. This research shows that women perceive more resources to cope with demands, in consequence, they perceive more well-being and better job performance. Regardless of gender, coordination, horizontal trust, vertical trust, and emotional competence stand as relevant resources for achieving healthy employees and healthy organizational outcomes. However, there are gender-specific predictors of healthy employees and healthy organizational outcomes, depending on gender.

**Discussion:**

Based on these results, gender-related recommendations for promoting specific resources (e.g., autonomy in women) and preventing specific demands (e.g., mobbing in men) may be suggested in organizational contexts.

## Introduction

In the 21st century, organizations are facing a reality marked by instability, crises and rapid changes in an environment, defined as VUCA (volatility, uncertainty, complexity, and ambiguity; Rajesh et al., 2019). In this context, all Human Resource Management (HRM) theories and models play a crucial role when focusing on improving the workers’ productivity. Moreover, the strategies that promote employees’ health and well-being have also demonstrated positive effects on productivity, emphasizing the narrow relationship between these variables (Guest, 2017). The job demands-resources theory provides a useful framework for investigating how various factors can influence these aspects (Demerouti et al., 2001). However, gaps remain in the literature regarding how these dynamics manifest and affect men and women differently (Powell and Greenhaus, 2010). Considering the VUCA reality, it is essential to analyze how job demands and resources impact men and women differently. Women, for instance, often face additional burdens such as family care and social pressure to fulfill traditional roles, which can affect their well-being and job performance (Eagly and Carli, 2007). Men, on the other hand, may encounter different expectations regarding their role as primary providers, which also generates stress and affects their performance (Duxbury et al., 2021). Moreover, a key characteristic of this context is the increase in global interconnections boosting diversity in the workplace, such as the entry of women in traditionally male-dominated sectors (Kaur and Arora, 2020). Therefore, the implementation of inclusive organizational practices promoting the organization’s performance and growth is required to properly manage this gender diversity (Dishon-Berkovits et al., 2024; Kaur and Arora, 2020). However, managing gender diversity does not always guarantee improved results in equality (Haile, 2012). Therefore, besides the implementation of these organizational practices, the organization is required to have sufficient resources at its disposal and to manage this diversity considering the gender differences (Gervais and Millear, 2024). In other words, gender diversity management must be done including the gender perspective. For practical purposes, this includes promoting resources that allow all employees to develop their skills and meet the specific requirements they may be faced with (Sturm, 2007). An example of how gender impacts on the requirements of the workforce was visible during the COVID-19 pandemic, when layoffs and work time reductions mainly fell on women, while these same women saw the gender-related demands increased (e.g., combine telework with domestic tasks and childcare), thereby affecting their well-being and performance (Cobos and Sánchez, 2020). Applying gender perspective in resource management to face the demands is key to improve organizational outcomes. Moreover, without gender perspective, organizational practices may perpetuate stereotypes affecting decisions regarding hiring, promotion, and rewarding, impacting negatively on occupational well-being (Sora et al., 2021). Given the importance of a gender perspective in organizational management, this study aims to analyze the predictors (resources, practices, and demands) with the highest impact on well-being and performance, thereby extending the HEalthy and Resilient Organizational Model from this perspective (Salanova et al., 2012, 2019). The novelty of the article is the following: (1) testing how gender influences the relationships between organizational and individual factors and employee and organizational outcomes, advancing the understanding of gender dynamics in the workplace; additionally, the use of a more heterogeneous sample compared to other studies focused on a single organization enhances the generalization of the conclusions; (2) provide HRM practitioners and policymakers with a framework to develop targeted, gender-sensitive strategies that better address the distinct needs of men and women, promoting overall well-being, inclusivity, and productivity and (3) focusing on these differentiated impacts, this study not only contributes to academic discourse but also offers a practical guide for HRM to create more equitable work environments, ultimately enhancing both employee well-being and organizational performance.

## Theoretical background

Several existing theoretical models can explain the differences in health, well-being and performance between men and women, as well as the effects of different genders sharing the same workspace (e.g., Person-Environment Fit Model; Edwards and Shipp, 2007). One of the oldest and most basic models in social psychology is the social identity theory (Tajfel and Turner, 1986). This theory supports that people are in search of a positive social identity through their affiliation to groups. In the context of gender differences at work, this theory could explain how comparisons and conflicts between men and women can have harmful effects on organizational outcomes, due to the rivalry and competition they generate. These comparisons can lead to tensions, discrimination, and a work environment that affects occupational well-being and health (Haile, 2012). However, this theory is limited when determining the concrete variables (such as demands and resources) that impact on health and organizational outcomes, since previous studies show that preventive work on these variables reduces conflicts between groups at work (Sureda et al., 2019). The Positive Occupational Health Psychology provides a comprehensive framework for the study of prevention and, specifically, the promotion of health and well-being in the work context (Bakker et al., 2012).

From this framework emerges the HERO model, stating that HEalthy and Resilient Organizations will cope more efficiently with the VUCA contexts (Salanova et al., 2019). This HERO model includes an organizational, integrative, and holistic perspective that points out the relevance of different organizational resources and practices allowing to meet job demands and to generate healthy employees, as well as to improve organizational outcomes (Salanova et al., 2019; Villarroel-Núñez et al., 2024). The model assumes that the promotion of resources (e.g., autonomy, coordination) and healthy organizational practices —HOPs— (e.g., communication practices, work-family balance practices, equality practices) increases workers well-being and improves their health (e.g., engagement) and performance (e.g., organizational commitment). It also enables organizations to emerge strengthened from crises and other common adverse situations in the VUCA context (Gil-Flórez et al., 2022; Gómez-Borges et al., 2022; Peñalver et al., 2023; Salanova et al., 2019). The inclusion of the gender perspective in the HERO Model and the inclusion of the demands as a factor in this article implies widening the model, allowing it to better adapt to the new VUCA contexts and the development of well-being and organizational outcomes in the companies.

Despite the relevance of considering gender perspective in the organizational context, there is a lack of studies allowing us to know which resources (job and personal), HOPs, and —in particular— demands are linked with well-being and performance in men and women. This model can be used from the gender perspective, meaning it can be used to detect gender-related key variables for well-being and performance. The HERO Model (Salanova et al., 2012) states the existence of three basic pillars or blocks of variables that are different, but nevertheless linked: (a) resources and healthy organizational practices (e.g., autonomy or work-family balance practices); (b) healthy employees (e.g., engagement, resilience); and (c) healthy organizational outcomes (e.g., in-role performance).

Originally (Salanova et al., 2012), the first block includes the two predicting factors of the healthy employees and organizational outcomes block: work resources and HOPs. On one hand, job resources are divided in task (e.g., autonomy) and group resources (e.g., coordination). Demerouti et al. (2001) define job resources as “the physical, psychological, organizational, or social work aspects that can reduce the demands of the job and the related physiological and psychological costs, be decisive in achieving the work results or stimulate personal growth, learning process, and well-being” (p. 2). Moreover, HOPs are considered “structural organizational resources,” together with the task resources (Salanova et al., 2019). Therefore, we can understand the HOPs as “planned human resource deployments and activities intended to enable an organization to achieve its goals” (Wright and McMahan, 1992, p. 298). However, the present study includes for the first time the role of job demands as predictors of healthy employees and organizational outcomes. The demands are job characteristics that will require physical and/or psychological (mental and emotional) effort, and therefore physical and/or psychological costs (Demerouti et al., 2001). These demands can be associated with the very nature of the task (a “boring” routine), to the interaction between the task and the worker (emotional dissonance or role ambiguity), or also the interactions between workers (mobbing). The second block of the HERO model includes the evaluation of *healthy employees’* factors. This block is comparable to that of psychosocial well-being. This well-being is defined by low levels of burnout or “syndrome of feeling burned out by the job” and high levels of self-efficacy, resilience and engagement (Salanova et al., 2019). Engagement can be defined as “a positive, affective-motivational state of fulfillment that is characterized by vigor, dedication, and absorption” (Schaufeli et al., 2002; p. 74). Previous studies have demonstrated that engagement in men and women and collective resilience are linked with better in-role and extra-role performance (Peñalver et al., 2023). All these psychosocial well-being variables —and engagement in particular— are mutually related with both job and personal resources (Xanthopoulou et al., 2009). Besides well-being, the HERO validation by Salanova et al. (2012) also includes personal resources —considered direct antecedents of both well-being and productivity— in the healthy employees’ block (Hernández-Vargas et al., 2014). These personal resources refer to “the personal skills, knowledge and characteristics that can be useful to face job demands” (Demerouti et al., 2001, p. 2).

The third block of the HERO Model gathers the *healthy organizational outcomes*: the intra-role (Goodman and Svyantek, 1999) and extra-role (fulfill tasks beyond what is expected in the workplace; Goodman and Svyantek, 1999) performance, as well as the employee’s commitment with the organization (Peñalver et al., 2023). This block is comparable to the concept of productivity which, besides being measured by means of scales, can also be measured by assessing the levels of Return-on-Investment (ROI) and work absenteeism (Salanova et al., 2019). However, these “objective indicators” of performance or productivity complicate the comparison between productivity levels of workers of different organizations when drawing conclusions regarding high external validity (Miguez and Menéndez, 2021).

Previous research provides evidence in favor of the HERO model: organizations that optimize and develop organizational resources and practices obtain healthier employees —meaning, with higher levels of well-being— who, consequently, achieve increased organizational outcomes (Salanova et al., 2012, 2019; Villarroel-Núñez et al., 2024). The present study allows us to recognize the most important predictors (demands, job and personal resources, and HOPs) of well-being and of the organizational outcomes from a gender perspective.

### Effects of resources and HOPs on healthy employees and healthy organizational outcomes

Previous research indicates that companies investing in improving job resources (e.g., good team spirit and high level of coordination; Salanova et al., 2012) impact positively: both on levels of resilience, efficacy, and engagement as on organizational outcomes. The same relationship applies to personal resources, both on healthy employees (e.g., mental and emotional competence; Tripiana and Llorens, 2015) as on organizational outcomes (e.g., vertical and horizontal trust; Guinot and Chiva, 2019; Salanova et al., 2021). Moreover, both types of resources (personal and job) can act as mediators of various indicators related with occupational well-being (Gómez-Borges et al., 2022, 2023). HOPs (i.e., work-family balance, mobbing prevention, psychosocial health and organizational communication) have demonstrated that they improve the productivity of the teams (Salanova et al., 2021). On the other hand, specific HOPs, aiming at mobbing prevention, promotion of communication and information, and skills and career development, allow the growth of a higher positive group affection (Peñalver et al., 2023) and collective empathy (Solares et al., 2016). However, hardly any studies are available on HOPs, specifically focusing on equality and gender diversity management. Therefore, these HOPs have been included in this study as a differentiated resource factor, putting to the test an extension of the model.

### Effects of demands on healthy employees and healthy organizational outcomes

Previous research has provided evidence that both employees’ health as their organizational outcomes are affected by the combination of high demands and lack of resources to meet them (Bakker and Demerouti, 2024), both at the individual (Salanova et al., 2019) and at the organizational level (Llorens et al., 2022). In particular, the perception of workers’ specific demands (i.e., mental overload) can determine the resources they will seek and use to meet them (i.e., mental competence). Possessing the necessary resources to meet a moderate demand allows to perceive this demand as a challenge instead of a threat, leading to better coping with it which, in turn, will result in healthier employees and organizational outcomes (Llorens et al., 2022; Salanova et al., 2019; Ventura et al., 2015). This is important since, as can be seen below, women tend to have a higher capacity to use resources, enabling them to better cope with demands (Cifre et al., 2011).

### Current study: HERO model with gender perspective

Previous literature exists, indicating specific gender-related differences in the variables that constitute the HERO model. In terms of resources and HOPs, some may be more necessary for a specific gender. For example, HOPs promoting autonomy have shown higher efficacy when it comes to promoting organizational outcomes in women (Gervais and Millear, 2014, 2024). With regard to demands, women and men may experience differences in demands, due to gender roles or to the position they occupy within the company (Cifre et al., 2000). For example, moderate role ambiguity can be positive for men’s job satisfaction, but not for women’s, due to their different motivations. In particular, role ambiguity only seems to be a predictor of job satisfaction in the male sample. The fact that role conflict also has a positive impact suggests that being submitted to role stress (role conflict and ambiguity) is motivating for men, since it brings along increased job activity (Cifre et al., 2000). On the other hand, men tend to perceive more mobbing and, since they possess a lower capacity to cope with it, this can have more negative consequences than in women (Cifre et al., 2011). Another example is the work–family conflict that men and women perceive differently, as the patriarchal system leads women to face more pressure due to caregiving duties, while men experience stress related to breadwinner expectations (Alcañiz, 2017). In terms of healthy employees, stress-derived symptoms are different between men and women in the same position (Ornek and Esin, 2020), since the job-related demands and the resources to meet them are different as well (Ornek and Esin, 2020). Finally, with regard to *organizational outcomes*, it must be noted that women tend to achieve higher scores in “job performance measures,” however, “ratings of promotion” are higher in men (Roth et al., 2012). Therefore, any measure aimed at improving organizational outcomes can be more effective if it is implemented from a gender perspective.

Despite recent studies concluding the impact of HERO variables such as resources (Gómez-Borges et al., 2022) and Healthy Organizational Practices (HOP) (Peñalver et al., 2023) across various contexts, including the validation of the HERO model from a multilevel perspective (Salanova et al., 2021), which highlight the effects on well-being and performance, very few studies have considered the gender perspective. This study allows putting to the test an extension of the HERO model by including this perspective. In this manner, it will constitute a key tool in the management of health and outcomes in organizations, especially those with high gender diversity, since it allows the study of gender-specific predictors for well-being and outcomes in organizations. In particular, the objective of this study is to identify the main predictors (resources, healthy organizational practices, and demands as novelty) for healthy employees and healthy organizational outcomes from a gender perspective. Considering this objective, the following hypothesis are expected:

*H1:* We expect to find significant gender-related differences in the perception of resources, HOPs, demands, healthy employees and organizational outcomes.

*H2:* We expect to find significant gender-related differences in the predictors (demands, resources and HOPs) for the block of healthy employees and organizational outcomes.

## Materials and methods

### Participants

The general study sample consisted of 3,863 employees from 8 Spanish companies (57% women and 43% men) from two different sectors (90% from the tertiary and 10% from the secondary sector). Missing value statistics were explored showing a significatively higher proportion of participants with missing data among men [proportion of men with missing data: 54.4%; proportion of women with missing data: 28.1%; *χ*^2^(1) = 132.97, *p* < 0.000]. The average of missing items per person was 3 for men against 1 for women. In order to keep a balanced sample between men and women, a multiple imputation method was performed through chained equations (Wulff and Jeppesen, 2017) using the R Studio MICE package (Van Buuren and Groothuis-Oudshoorn, 2011). After the imputation, the final sample consisted of 2,128 employees (70.3% women, 30.5% between 36 and 45 years old) representing various economic sectors, with 84% affiliated with the tertiary sector (specifically, hospitals; 80.4% women) and the remaining 16% with the secondary sector (100% men). Table 1 shows the results of the description of women and men taking into account the age.

**Table 1:** Description of women and men by age and missing data.

	Women				Men			
Age group	18–35	36–45	46–55	56+	18–35	36–45	46–55	56+
*n*	448	516	334	198	182	226	139	85
Missing values	101 (22.55%)	158 (30.62%)	104 (31.14%)	57 (28.79%)	895 (51.65%)	126 (55.75%)	80 (57.55%)	44 (51.77%)
Average missing values per person	1	1	1	1	3	3	3	2

### Procedure

Data were collected from participants through an online survey uploaded onto the Qualtrics platform. A survey invitation was sent via email. The information about the project was presented in three stages for all organizations. First, an introductory meeting took place with the CEOs; second, a meeting involving managers and the HR department. In the third meeting, the data collection process was planned. The HR departments arranged suitable locations and groups for employees to complete the questionnaires in shifts. Prior to answering the questionnaires, anonymity was ensured for all participants and the informant consent agreement was provided for signature on voluntary basis.

### Measurement instruments

*Healthy Organizational Practices* were assessed at the organizational level with 19 items included in the HERO-CHECK questionnaire (Salanova et al., 2012, 2019). Subsequently, 8 healthy organizational practices (HOPs) —related to gender and other diversities— were considered in the analyses, each with one item: work-family balance, equity, equal opportunities, maternity/paternity, skills and talent, leadership equality, culture diversity, and training diversity. We used a 7-point Likert-type scale ranging from 0 ‘never’ to 6 ‘always’. An example of item is ‘[*In the last year, practices and strategies have been introduced in this organization in order to expand the requirements of current legislation to enjoy maternity/paternity benefits (e.g., low extension, flexible hours)*]’ (𝞪 = 0.95; 𝟂 = 0.95).

*Job and personal resources* were assessed with 9 items included in the HERO-CHECK questionnaire (Salanova et al., 2012, 2019). These items were: autonomy, feedback, supportive climate, coordination, mental competence, emotional competence, positive leadership, vertical trust, and horizontal trust. Respondents answered using a 7-point Likert-type scale (from 0 ‘strongly disagree’ to 6 ‘strongly agree’). An example of item is ‘*Degree to which they have sufficient control to decide the tasks they will carry out during the day, the number of tasks, the order in which they will be carried out, and the moment they will start and/or finish them*’ (𝞪 = 0.88; 𝟂 = 0.91).

*Job demands* were assessed with eight items, based on the HERO-CHECK questionnaire (Salanova et al., 2019; Villarroel-Núñez et al., 2024). These items were the following: quantitative overload, mental overload, emotional overload, role ambiguity, role conflict, routine, mobbing, and emotional dissonance. Respondents answered using a 7-point Likert-type scale (from 0 ‘strongly disagree’ to 6 ‘strongly agree’). An example of item is ‘*Degree to which the amount of work to be done “overwhelms” them, either due to lack of time or due to excess tasks*’ (𝞪 = 0.81; 𝟂 = 0.81).

*Healthy employees* were assessed with 4 items, based on the HERO-CHECK questionnaire (Salanova et al., 2012, 2019). These items were: self-efficacy, work engagement, resilience, and burnout. Respondents used a 7-point Likert-type scale (from 0 ‘strongly disagree’ to 6 ‘strongly agree’). An example of item is ‘*Degree to which they believe in the abilities of their work team to carry out tasks successfully despite obstacles*’ (𝞪 = 0.77; 𝟂 = 0.79).

*Healthy organizational outcomes* were assessed using three items from the HERO-CHECK questionnaire (Salanova et al., 2012, 2019). These items were: in-role performance, extra-role performance, and commitment. Respondents used a 7-point Likert-type scale (from 0 ‘strongly disagree’ to 6 ‘strongly agree’). An example of item is ‘*Degree to which the tasks of their work are carried out and fulfilled, those that are prescribed in their employment contract*’ (𝞪 = 0.46; 𝟂 = 0.54). The item of extra-role performance was deleted in order to improve the internal consistency of the scale (𝞺 = 0.44, *p* < 0.001).

### Data analysis

Descriptive data analysis (means, standard deviations per gender) was performed using R 1.4.2 (R Core Team, 2021). Scale reliability was estimated with Cronbach’s alpha (𝞪) and MacDonald’s Omega (𝟂) total using Psych R package (Revelle, 2017). Gender differences were tested on each item. First, the *F-*test for equality of variances for checking homoscedasticity among genders was used. Depending on the homoscedasticity results, *t* test analysis was performed for gender groups (with Welch approximation to the degrees of freedom if variances are not equal). Due to the large sample size, normality was assumed on the distribution without compromising results (Pardo Merino and San Martín, 2010).

To ensure the robustness and validity of the theoretical model, a Confirmatory Factor Analysis (CFA) was conducted using the lavaan R package (Rosseel, 2012). The CFA was employed to verify the factor structure of the proposed model and assess the potential influence of common method variance through Harman’s single-factor approach (Podsakoff et al., 2003). The estimation method utilized was Maximum Likelihood with Satorra-Bentler correction (MLMV), which provides more reliable results, especially in situations where data may not perfectly conform to normality (Maydeu-Olivares, 2017). Additionally, to ensure the independence of variables, collinearity among items was assessed using the Variance Inflation Factor (VIF), following guidelines by Kock (2015).

The CFA results were tested by considering three absolute goodness-of-fit indices to evaluate the goodness-of-fit of the models: (1) the chi-squared (*χ*^2^) goodness-of-fit statistic, (2) the Root Mean Square Error of Approximation (RMSEA), and (3) the standardized root mean square residual (SRMR); and two relative goodness-of-fit indices: (1) Comparative Fit Index (CFI) and (2) Tucker-Lewis Index (TLI, also called the Non-Normed Fit Index). For RMSEA and SRMR, values smaller than 0.05 indicated an excellent fit, 0.08 indicated an acceptable fit, and values greater than 0.10 led to model rejection (Browne and Cudeck, 1993). Regarding the relative fit indices, values greater than 0.90 indicated a good fit (Hu and Bentler, 1999).

Finally, a multigroup structural equation model (SEM) was tested using Lavaan R package (Rosseel, 2012) to determine which predictors (i.e., *Healthy Organizational Practice*s, *Job demands* and *Job Resources*) are related to *Healthy employees* and *Healthy organizational outcomes*. *Healthy employees* and *Healthy organizational outcomes* were included as latent factors, operationalized by four (self-efficacy, engagement, resilience, and burnout) and two (i.e., in-role performance, and commitment) items, respectively (Salanova et al., 2012; Villarroel-Núñez et al., 2024). Predictor items were included as exogenous observed variables in the model. Maximum likelihood with Satorra-Bentler correction (MLMV) was used as an estimation method (Maydeu-Olivares, 2017). Absolute and relative indexes of goodness-of-fit (GoF) (Marsh et al., 1996) were used to test the adequacy of the model: chi square statistic, Root Mean Square Error of Approximation (RMSEA), Standardized Root Mean Square Residual (SRMR), Tucker-Lewis Index (TLI), and Comparative Fit Index (CFI). Values smaller than 0.08 for RMSEA and SRM, and greater than 0.90 for TLI and CFI indicate an acceptable fit (Marsh et al., 2004).

To test the invariance of the proposed model across genders, we followed the free baseline approach (Stark et al., 2006) that compares models with different levels of constraints for parameter estimation between groups and a baseline model with no constraints (Bentler, 2000). First, we tested measurement invariance following Meredith’s classification (1993) (Meredith, 1993). Subsequently, we compared the unconstrained baseline model (M1) with (a) a model with restricted factor loadings (M2) to test weak factorial invariance; (b) a model with constrained factor loadings and intercepts (M3) that would support strong factorial invariance; (c) a model with constraints in factor loadings, intercepts, and measure residuals (M4), testing strict invariance; (d) a model with constraints in factor loadings, intercepts and measure residuals, and factor means (M5) for testing structural invariance and finally, (e) a model with constraints in factor loadings, intercepts, residuals and regressions across gender groups. Strong factorial invariance is considered sufficient for testing predictions in latent variables across groups (Newson, 2023).

To evaluate the magnitude of change between the baseline model and the constrained ones, the chi-square difference test between reproduced models was estimated, as well as the increments in RMSEA and CFI indexes (Chen, 2007; Hu and Bentler, 1999). Low changes in CFI, TLI, RMSEA, and SRMR indexes (usually accepted |*Δ* < 0.01|) indicate parameter invariance among groups (Cheung and Rensvold, 2002).

## Results

### Descriptive, reliability, collinearity and validity analysis

Descriptive statistics, statistical differences among genders, and reliability analysis results are displayed in Table 2 to test Hypothesis 1. Cronbach’s alpha and McDonalds’ omega values show excellent results (above 0.70) (Campo-Arias and Oviedo, 2008).

**Table 2:** Descriptive statistics, gender differences per item and reliability results.

Scale	Item	Women	Men	Women	Men	*t* (*p*-value)	Scale *α*	Scale 𝟂
		Mean	Mean	SD	SD			
Job demands	Quantitative overload	4.35	4.32	1.39	1.39	−0.44	0.81	0.81
	Mental overload	5.13	5.19	1.26	1.26	0.91		
	Emotional overload	4.80	4.61	1.38	1.38	−2.8***		
	Role ambiguity	2.45	2.65	1.34	1.34	3.13***		
	Role conflict	2.77	2.87	1.37	1.37	1.57		
	Routine	3.20	3.61	1.52	1.52	5.92***		
	Mobbing	2.00	1.99	1.32	1.32	−0.23		
	Emotional dissonance	3.27	3.21	1.58	1.58	−0.76		
Job and personal resources	Autonomy	5.45	5.19	1.54	1.54	−3.68***	0.88	0.91
	Feedback	5.54	5.13	1.36	1.36	−6.53***		
	Supportive climate	5.99	5.62	1.29	1.29	−6.27***		
	Coordination	5.99	5.82	1.14	1.14	−3.2***		
	Positive leadership	5.91	5.45	1.42	1.42	−7.1***		
	Horizontal trust	6.08	5.91	1.02	1.02	−3.51***		
	Vertical trust	5.68	5.34	1.33	1.33	−5.48***		
	Mental competence	5.87	5.77	1.06	1.06	−2.00*		
	Emotional competence	5.89	5.83	1.02	1.02	−1.36		
Healthy organizational practices	Work-family balance practices	4.68	3.44	2.28	2.28	−12.02***	0.95	0.95
	Equity	4.57	3.33	2.29	2.29	−11.99***		
	Equal opportunities	5.56	4.14	2.55	2.55	−12.87***		
	Maternity/paternity	5.38	3.92	2.54	2.54	−13.07***		
	Skill and talent	5.67	4.17	2.55	2.55	−13.75***		
	Leadership equality	5.19	3.75	2.37	2.37	−13.71***		
	Culture diversity	5.39	3.88	2.36	2.36	−14.60***		
	Training diversity	4.72	3.32	2.35	2.35	−13.17***		
Healthy employees	Self-efficacy	6.05	5.83	1.06	1.06	−4.50***	0.77	0.79
	Resilience	5.70	5.51	1.19	1.19	−3.42***		
	Engagement	5.85	5.58	1.21	1.21	−4.93***		
	Burnout	2.34	2.64	1.50	1.50	4.38***		
Healthy organizational outcomes	In-role performance	6.42	6.16	1.04	1.04	−5.46***	𝞺 = 0.44***	
	Commitment	6.06	5.89	1.26	1.26	−2.79**		

*p < 0.05; **p < 0.01; ***p < 0.001.

Significant differences between genders were found for most items. In terms of demands, women showed significantly higher emotional overload, while men experienced higher role ambiguity and routine. Women showed statistically higher levels of resources and organizational practices, except for emotional competence. In addition, scores for wellness and performance items were higher for women than for men. In general terms, these results confirm our Hypothesis 1.

Harman’s single-factor test result indicates that the single-factor solution does not explain the variance in the data [*χ*^2^(495) = 18106.39, *p* < 0.000; CFI = 0.59; TLI = 0.56; RMSEA = 0.13; SRMR = 0.13], so common method variance is not a serious problem in this study [∆*χ*^2^ (10) = 2727.6, *p* < 0.01; ∆CFI = 0.28; ∆TLI = −0.30; ∆RMSEA = −0.06; ∆SRMR = −0.06]. Additionally, VIF results were below the 3.3 cut-off value (Kock, 2015), except for the items evaluating practices in equal opportunities (VIF = 4.295), maternity/paternity (VIF = 3.547), skill and talent (VIF = 5.223), leadership equity (VIF = 4.476), and culture diversity (VIF = 5.091). However, only culture and diversity slightly exceeded the alternative threshold of 5 proposed by Ringle et al. (2015).

**Figure 1:**
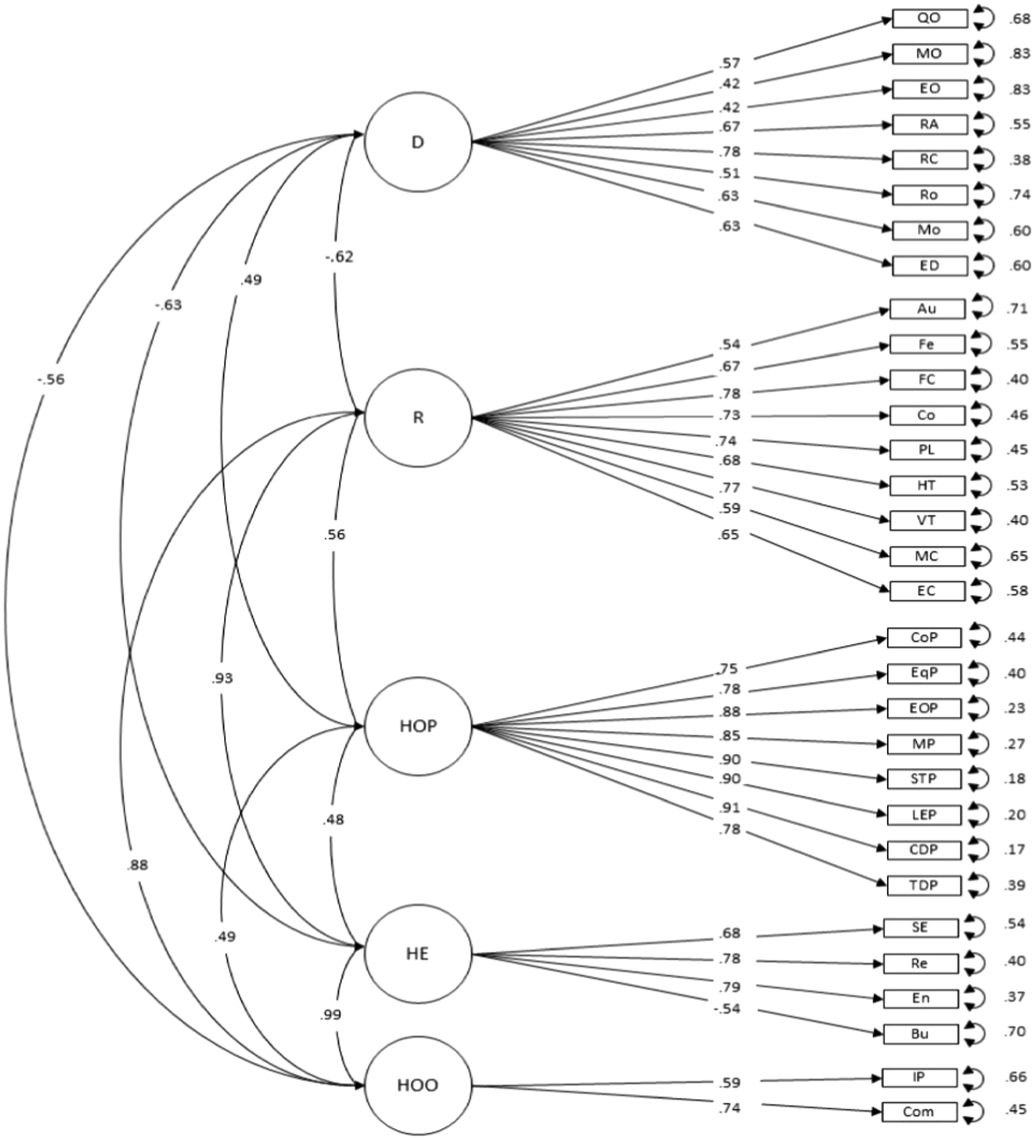
CFA Results. D = Job demands [quantitative overload (QO), mental overload (MO), emotional overload (EO), role ambiguity (RA), role conflict (RC), routine (Ro), mobbing (Mo), emotional dissonance (ED)]; R = job and personal resources [autonomy (Au), feedback (Fe), supportive climate (SC), coordination (Co), positive leadership (PL), horizontal trust (HT), vertical trust (VT), mental competence (MC), emotional competence (EC)]; HOP = healthy organizational practices [work-family balance (WFB), equity (Eq), equal opportunities (EOpp), maternity/paternity (MP), skills and talent (STP), leadership equality (LEP), culture diversity (CDP), training diversity (TDP)]; HE = healthy employees [self-efficacy (SE), work engagement (WE), resilience (Re), burnout (Bu)]; HOO = Healthy Organizational Outcomes [in-role performance (IP), commitment (Com)].

The results of the CFA factor model show that the model with 5 dimensions (i.e., job demands, job and personal resources, organizational practices, healthy employees and healthy organizational outcomes) has an acceptable fit (RMSEA and SRMR values <0.08) although the relative fit indices did not reach the 0.9 cutoff value, but were above 0.85 (CFI = 0.88; TLI = 0.87; RMSEA = 0.07). All items showed loadings over 0.40, which has been considered the cutoff value of the contribution of each item to the latent factor variability (Guadagnoli and Velicer, 1988; see Figure 1).

### SEM and invariance tests

**Figure 2:**
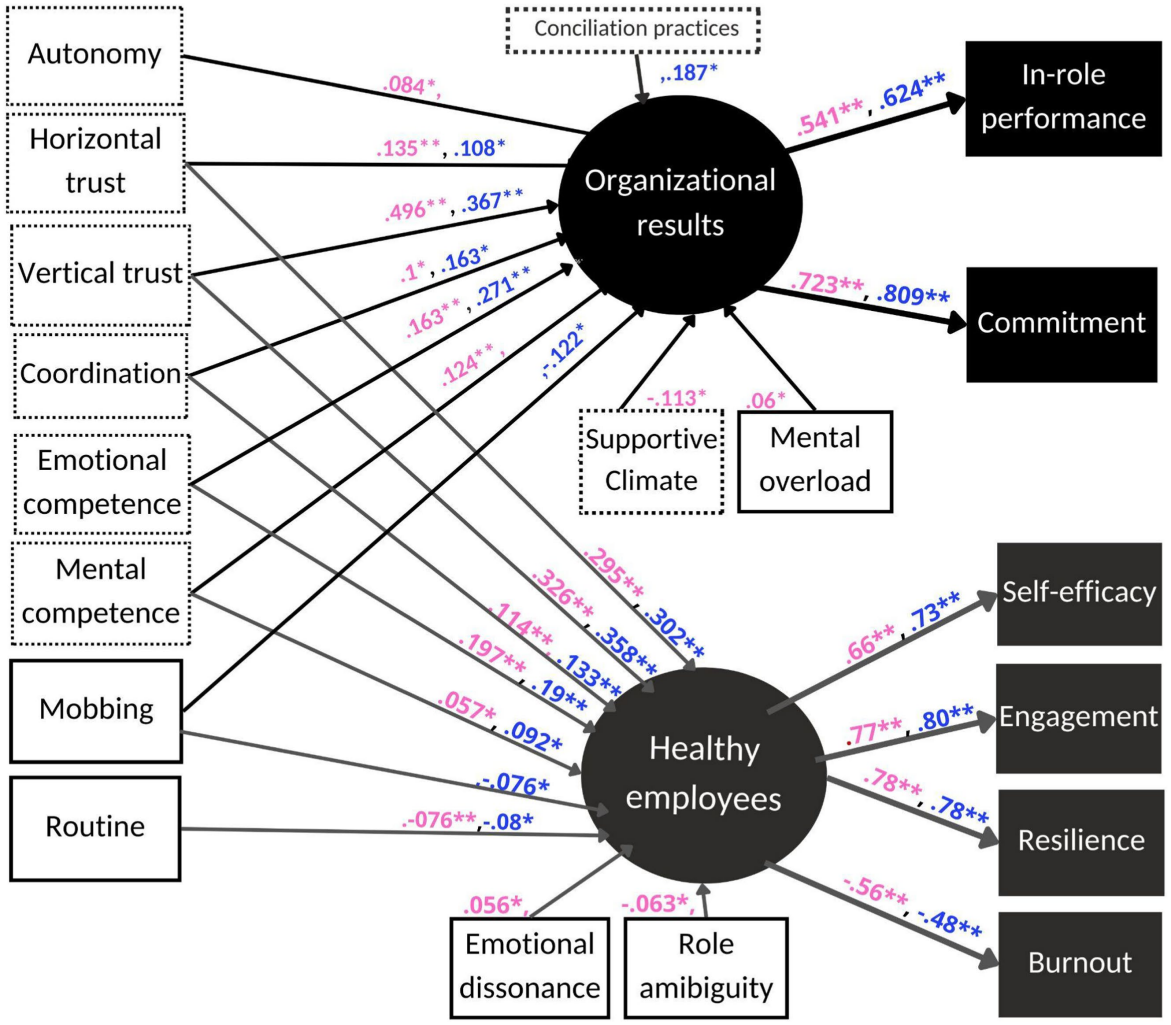
Gender-based multigroup SEM. *p < 0.05, **p < 0.01. For the scores, a comma is placed immediately after the number for women and immediately before the number for men. Additionally, pink represents women and blue represents men. The predictor variables for resources and practices are indicated with dotted lines, while demands are represented with solid lines.

The GoF for models with different constraints are displayed in Table 3. The baseline model (M1) showed a good fit (CFI = 0.874; RMSEA =0.060) that indicates the adequacy of the model. All items considered for the measurement model had significant factor loading in their factor (i.e., healthy employee and healthy organizational outcomes) in both groups. Standardized factor loadings were above 0.4 —which is generally considered a cut-off value (Guadagnoli and Velicer, 1988)— for all items (Figure 2).

**Table 3:** Goodness-of-fit Indexes and comparison for Multigroup SEM.

Models	*χ*^2^	df	*p*	RMSEA	CFI	TLI	SRMR	∆*χ*^2^	∆df	∆RMSEA	∆CFI	∆TLI	∆SRMR
M1	1.114.256	216	0.000	0.063	0.890	0.832	0.027						
M2	1.127.862	220	0.000	0.062	0.889	0.833	0.028						
M3	1.148.451	224	0.000	0.062	0.887	0.833	0.028						
M4	1.211.203	230	0.000	0.063	0.880	0.828	0.029						
M5	1.222.442	232	0.000	0.063	0.879	0.828	0.030						
M6	1.229.354	274	0.000	0.057	0.883	0.859	0.030						
Diff. M1- M2								−13.606	−4	0.001	0.001	−0.001	−0.001
Diff. M1-M3								−34.195	−8	0.001	0.003	−0.001	−0.001
Diff. M1- M4								−96.947	−14	0.000	0.010	0.004	−0.002
Diff. M1- M5								−108.186	−16	0.000	0.011	0.004	−0.003
Diff. M1- M6								−115.098	−58	0.006	0.007	−0.027	-0.003

*p < 0.5; **p < 0.01; ***p.001 *χ*^2^ = Chi-square; df = degree of freedom; RMSEA = Root Mean Square Error of Approximation; CFI = Comparative Fit Index; TLI = Tucker-Lewis Index; SRMR = Standardized Root Mean Square Residual; Diff.;∆ = differences; M1 (Baseline Model): no restrictions across gender groups; M2: constraints applied to factor loadings across gender groups; M3: constraints applied to factor loadings and intercepts across gender groups; M4: Constraints applied to factor loadings, intercepts, and residual variances across gender groups; M5: constraints applied to factor loadings, intercepts, residuals, and factor means across gender groups; M6: constraints applied to factor loadings, intercepts, residuals, and regressions across gender groups.

Regarding measurement invariance across genders, the results confirm strict invariance, that is, there is no variability in factor loadings, intercepts, and residuals among gender groups. Results of the chi-square test show that all models differ significantly from the baseline model. Absolute increments in GoF between M1 and M2, M3 and M4 do not reach the 0.01 cut-off value. Regarding the difference between M1 and M5, CFI decrease is above 0.01. Considering the sensitivity of chi-square tests, we conclude that the proposed model has strict invariance. This level of invariance allows comparisons between regression coefficients (see Table 3).

Standardized beta scores for SEM paths between associated z-scores and *p*-values for both latent factors are displayed in Table 4 to test Hypothesis 2. Significant regressions in the women’s group for *healthy employee* were found in *role ambiguity* (*β* = −0.063, *p* < 0.01), *routine* (*β* = −0.076, *p* < 0.001), *emotional dissonance* (*β* = −0.056, *p* < 0.01), *coordination* (*β* = 0.114, *p* < 0.001), *horizontal trust* (*β* = 0.295, *p* < 0.001), *vertical trust* (*β* = 0.326, *p* < 0.001), *mental competence* (*β* = 0.057, *p* < 0.01), and *emotional competence* (*β* = 0.197, *p* < 0.001). For men, significative regressions were found in *routine* (*β* = −0.08, *p* < 0.01), *mobbing* (*β* = −0.076, *p* < 0.05), *coordination* (*β* = 0.133, *p* < 0.001), *horizontal trust* (*β* = 0.302, *p* < 0.001), *vertical trust* (*β* = 0.358, *p* < 0.001), *mental competence* (*β* = 0.092, *p* < 0.05), and *emotional competence* (*β* = 0.19, *p* < 0.001).

**Table 4:** SEM standardized coefficients for latent factors.

	Healthy employee				Healthy organizational outcomes			
	Women		Men		Women		Men	
	*β*	*z* (p)	*β*	*z* (p)	*β*	*z* (p)	*β*	*z* (p)
Quantitative overload	−0.021	−1.012	−0.017	−0.507	−0.005	−0.162	0.033	0.759
Mental overload	0.014	0.687	0.035	1.00	0.065*	2.155	0.031	0.787
Emotional overload	0.025	1.201	0.008	0.258	0.003	0.114	0.038	0.943
Role ambiguity	−0.063	−2.582*	−0.055	−1.681	−0.049	−1.284	0.039	0.844
Role conflict	0.002	0.088	−0.019	−0.491	−0.025	−0.654	−0.067	−1.298
Routine	−0.076	−3.85***	−0.08	−3.00*	−0.021	−0.753	0.043	1.013
Mobbing	−0.039	−1.716	−0.076	−2.00*	−0.057	−1.596	−0.129	−2.835**
Emotional dissonance	−0.056	−2.787*	0.032	0.975	−0.016	−0.53	0.024	0.545
Autonomy	0.039	1.93	0.036	1.00	0.078	2.315*	0.024	0.6
Feedback	0.029	1.384	0.013	0.345	−0.024	−0.737	0.034	0.67
Supportive climate	0.025	0.898	0.021	0.45	−0.115	−2.518*	0.061	1.032
Coordination	0.114	4.304***	0.133	3.00***	0.101	2.572*	0.161	2.663**
Positive leadership	−0.036	−1.33	−0.044	−0.871	−0.012	−0.258	−0.059	−0.974
Horizontal trust	0.295	8.605***	0.302	6.00***	0.139	3.224***	0.111	2.233*
Vertical trust	0.326	9.664***	0.358	7.00***	0.504	5.411***	0.382	4.628***
Mental competence	0.057	2.454*	0.092	2.3*	0.122	3.105***	0.056	1.084
Emotional competence	0.197	7.049***	0.19	4.00***	0.166	3.557***	0.271	3.97***
Work-family balance	−0.015	−0.687	0.005	0.092	0.029	0.794	0.183	2.686**
Equity	0.021	0.89	0.071	1.00	−0.013	−0.36	−0.025	−0.359
Equal opportunities	0.00	0.012	−0.086	−0.993	0.066	1.462	−0.058	−0.448
Maternity/paternity	0.02	0.806	0.021	0.365	0.001	0.028	0.132	1.522
Skill and talent	−0.033	−0.991	−0.062	−0.651	−0.016	−0.311	−0.185	−1.614
Leadership equality	0.022	0.755	0.036	0.575	0.026	0.541	−0.034	−0.388
Culture diversity	0.032	1.006	−0.031	−0.321	0.051	1.008	0.045	0.398
Training diversity	0.03	1.313	−0.022	−0.484	0.029	0.808	−0.054	−0.893

*β* coefficients are standardized, *p < 0.05; **p < 0.01; ***p < 0.001.

These results suggest that *routine*, *coordination*, *horizontal trust*, *vertical trust*, *mental competence*, and *emotional competence* have a significant influence on healthy employees for both men and women. As expected, all significant regressions related to job demands (i.e., *role ambiguity, routine, mobbing, and emotional dissonance*) were negatively related to *healthy employee*, whereas the variables related to *job and personal resources* (i.e., *coordination, horizontal trust, vertical trust, mental competence*, and *emotional competence*) had a positive value. Diversity-related *healthy organizational practices* were not significantly linked to healthy employees in any group.

Regarding significant beta scores in *healthy organizational outcomes* in the women’s group, significant values were found for *mental overload* (*β* = 0.065, *p* < 0.05), *autonomy* (*β* = 0.078, *p* < 0.01), *supportive climate* (*β* = − 0.115, *p* < 0.01), *coordination* (*β* = 0.101, *p* < 0.01), *horizontal trust* (*β* = 0.139, *p* < 0.001), *vertical trust* (*β* = 0.504, *p* < 0.001), *mental competence* (*β* = 0.122, *p* < 0.05), and *emotional competence* (*β* = 0.166, *p* < 0.001). For the men’s group, significant regressions were found in *mobbing* (*β* = − 0.129, *p* < 0.01), *coordination* (*β* = 0.161, *p* < 0.01), horizontal *trust* (*β* = 0.111, *p* < 0.05), *vertical trust* (*β* = 0.382, *p* < 0.001), *emotional competence* (*β* = 0.271, *p* < 0.001), and *work-family balance practices* (*β* = 0.183, *p* < 0.001).

For both men and women, *coordination*, *horizontal trust*, *vertical trust*, and *emotional competence* had a significant weight in healthy organizational outcomes. Against expectations, *mental overload* was positively related with *healthy organizational outcomes* in the women’s group, whereas *supportive climate* was negatively related. *Coordination, horizontal trust, vertical trust*, and *emotional competence* are significant and positively related for both men and women as for *healthy employees* and *healthy organizational outcomes*. For the women’s group, *mental competence* showed significant scores in both latent factors. For the men’s group, *mobbing* was significant and negatively related with *healthy employees* and *healthy organizational outcomes*. These results partially support Hypothesis 2.

## Discussion

The general objective was to expand knowledge on key drivers of healthy employees and organizational outcomes, extending the HERO Model by including gender perspective and the role of job demands as a novelty. To achieve this, confirmatory factor analysis (CFA) was employed to validate the predictors (job demands, and resources and healthy organizational practices) of two latent factors (Healthy Employees and Healthy Organizational Outcomes). This approach highlights the differential impacts of these three predictors for men and women. Following the CFA, an exploratory SEM model was conducted. Results of the multigroup SEM analysis show a good fit of the HERO model and support the existence of configural invariance among gender groups, thus extending the original HERO model by integrating job demands and gender perspective. We confirmed through CFA that there are five factors and subsequently demonstrated which are the most important specific variables within each factor in the prediction of healthy employees and healthy organizational outcomes among genders. Results align with Hypothesis 1: women show more resources (except emotional competence, where differences are not significant), higher HOPs, higher organizational results and healthy employees compared to men. Moreover, women report significantly higher emotional overload, lower role ambiguity, and routine.

Based on prior studies, it cannot be concluded that women experience a greater intensity of demands compared to men; however, there are clear differences in the types of demands imposed by work environments (Alcañiz, 2017; Cifre et al., 2011; Cifre and Vera, 2019; Payá and Beneyto, 2019). Moreover, with the exception of the emotional variable, women perceive higher levels of resources (Cañavate et al., 2023); therefore, it is logical and consistent with the HERO model that these are reflected in better coping with demands which, in turn, has a positive impact on their healthy organizational outcomes and healthy employees.

This research validates the extension of the HERO model by providing evidence on the most significant demands, resources, and practices to predict healthy employees and organizational results from a gender perspective. Specifically, we can confirm Hypothesis 2 positing that there are gender-based differences in predictors of healthy employees and results. Women demonstrate more predictors —including demands and resources— within the healthy employee and organizational outcomes. However, the HOPs were not predictive in either gender. Previous studies (Salanova et al., 2012, 2021; Tripiana and Llorens, 2015; Villarroel-Núñez et al., 2024) have highlighted certain resources and demands. However, our findings reveal that coordination, horizontal trust, vertical trust, and emotional competence emerge as the most significant resources for achieving healthy employees and results, regardless of gender. In terms of healthy employees, mental competence is identified as a relevant resource, while routine emerges as a relevant demand for both men and women. Additionally, role ambiguity and emotional dissonance only demonstrate significant beta scores in women, possibly due to gender-dependent coping mechanisms (Mulder et al., 2017). Regarding healthy organizational outcomes, autonomy and mental competence only show significance in women (consistent with previous studies, Gervais and Millear, 2014, 2024). In addition, this aligns with previous studies showing that women seek companies that are more committed to flexibility and proper diversity management (McKinsey and Company, 2023). Moreover, regarding mobbing, this aspect only predicts less healthy employees and outcomes in men, suggesting potential barriers in terms of communication or help-seeking among men experiencing stress (Galdas et al., 2005).

These results underline an important finding —aligned with previous research (Cañavate et al., 2023)— indicating that women not only exhibit more resources and demands affecting occupational well-being and organizational outcomes, but also possess more resources to cope with demands, thereby enhancing well-being and performance as measured by the HERO. However, further analysis is needed with the effects of two “against expectations”-variables: in the women’s group, mental overload was positively related with healthy organizational outcomes, whereas supportive climate was negatively related.

First, mental overload: Despite being the lowest scoring factor in explaining healthy organizational outcomes in women (6%), there is a significant positive and counterintuitive relationship between these two variables that can be explained by previous studies. Demands such as time pressure and overload can increase workers’ activity and motivation, positively affecting well-being and development (Crawford et al., 2010; Bakker and Sanz-Vergel, 2013; Ventura et al., 2015). This occurs especially when they are perceived as challenging demands. To transform a threatening demand into a challenging demand, it is crucial to perceive adequate resources to cope with it (Salanova et al., 2019). In this study, mental competence (the key resource to face mental overload) predicts better healthy organizational outcomes only in women, who also show significantly higher scores in mental competence compared to men. Additionally, despite their stress potential, challenging demands can drive employees to acquire more resources and invest more effort (Webster et al., 2010). Therefore, this significant positive relationship between mental overload and healthy organizational outcomes in women can be attributed to their higher mental competence, which helps them perceive mental overload as a challenge rather than a threat, thus enhancing their well-being and performance.

Second, supportive climate: The impact of a supportive climate on women —compared to men— has been a topic of interest for decades (Fusilier et al., 1986). This interest has grown with the increasing presence of women in traditionally male-dominated jobs. Certain types of social support can negatively affect performance and health, e.g., when this support highlights workplace stress, makes the recipient feel inadequate, or is unwanted (Beehr et al., 2010; Deelstra et al., 2003). Women are more likely to receive this inadequate support, especially in the form of “mansplaining,” where men provide unsolicited explanations that undermine women’s abilities (Jack et al., 2021). All this could help to understand why —in the case of women— a supportive climate is related to lower healthy organizational outcomes.

In the present study, HOPs predict healthy employees, but only work-family balance practices predict healthy organizational outcomes, moreover, only in men. Understanding this requires considering gender roles in work-family issues. For example, women’s use of work-family coping strategies is more associated with work–family conflict and enrichment than men’s (Matias and Fontaine, 2015). This disparity is due to the double burden women face, managing both job and domestic responsibilities, unlike men. Men who use paternity leave benefit from genuine rest and return to work rejuvenated. Conversely, women’s use of these policies might be misinterpreted as a lack of career commitment, hindering their progression (McKinsey and Company, 2023). The study’s findings on HOPs are consistent with previous research showing limited effectiveness of these practices in improving organizational outcomes (Haile, 2012).

The Haile study grouping HRM practices into three categories —gender equality policies, equal opportunities training, and monitoring gender equality provisions— further supports this. It highlights the low effectiveness of HR gender equality practices in improving organizational outcomes, in line with our study’s findings (Farré, 2013). Some HR practices benefit the organization more than the employee’s well-being, especially gender practices that are implemented as a formality (Ogbonnaya et al., 2017). Besides this, some benefits of HR practices are often skewed in favor of the organization at the expense of employees’ well-being (Ogbonnaya et al., 2017). Probably, this is more likely to occur in gender practices that are carried out as a matter of policy.

### Theoretical implication

In conclusion, our results support the effectiveness of promoting specific resources and prevent specific demands to build positive organizations among genders. Concretely, horizontal and vertical trust, emotional and mental competence, coordination, and routine predict healthy employees and/or results for both men and women. In terms of demands, only mobbing is negatively related to healthy employees and results in men, while only role ambiguity and emotional dissonance is negatively related with healthy employees in women. Furthermore, autonomy and mental competence predict healthier organizational outcomes in women (not in men), while they also perceive more resources (except emotional competence) and HOPs.

In addition, the study unveils three surprising findings:

Firstly, mental overload is found to be positively associated with healthy organizational outcomes in women. This underscores the importance of perceived competence in managing challenging demands, which may motivate employees to excel, potentially enhancing commitment and organizational performance. Secondly, supportive climate shows gender differences in the perception of support and the prevalence of “mansplaining” highlights the importance of fostering a real supportive environment —especially for women— to mitigate adverse effects on performance and well-being. Thirdly, only work-family balance practices predict healthy organizational outcomes, and only in men. This underscores the need for gender-sensitive practices that address work-family dynamics and institutional barriers to foster equity and productivity. One key lesson is that being competitive and surviving successfully in a VUCA context could not only depend on promoting resources to workers to cope with the demands, but also on doing so in a gender-sensitive manner.

### Practical implications

The practical takeaway from the research for sample firms revolves around the notion of investing in practices to promote job resources—such as coordination—and personal resources—including horizontal and vertical trust, as well as emotional and mental competence to foster healthier employees and yield positive outcomes for both men and women. Besides this, organizations must consider the more complex theoretical implications of this research, which show the different importance of demands and resources for the well-being and performance of each gender. Given that women experience greater emotional dissonance and perceive less emotional competence, interventions are urgently needed to provide them with the ability to cope with the specific burdens they may feel, such as emotional intelligence training sessions. Additionally, providing women with greater autonomy could help them address demands that affect their health and well-being, such as role ambiguity. On the other hand, HRM must pay more attention to prevent mobbing in men, gender-sensitive interventions breaking down gender stereotypes to stimulate men in the communication of vulnerability can help (Staiger et al., 2020).

In addition, the fact that work-family balance practices only positively affect the performance of men can be addressed by emphasizing the need for effective supervisor support and organizational cultures that genuinely value and support the use of work-family balance practices without gender bias (McKinsey and Company, 2023). In other respects, the fact that other HRM practices related to gender equality and diversity are not resulting in significant effects on well-being and labor productivity brings along the urgent need for CEOs and politicians to test the effectiveness of the practices implemented and review the sums invested in them to improve their effects, especially in the case of women.

### Limitations and future research

The present study has some limitations. The first is that all data were obtained through cross-sectional self-reports. Future studies could focus on longitudinal measures to test their stability. Secondly, an important limitation of this study is related to the significant number of missing values in our data set. A notably higher proportion of missing data was observed among male participants (54.4%) compared to female participants (28.1%), as indicated by a significant chi-square test result [*χ*^2^(1) = 132.97, *p* < 0.000]. This disparity in missing data could introduce bias and affect the reliability of the findings. Although a multiple imputation method was employed to address this issue and maintain a balanced sample between genders, the potential impact of the missing data on our results cannot be entirely ruled out. However, the use of advanced imputation techniques has likely minimized any distortions, allowing for more robust and reliable conclusions despite these challenges.

## Data Availability

The raw data supporting the conclusions of this article will be made available by the authors, without undue reservation.
